# On the Influence of the Sample Absorptivity when Studying the Thermal Degradation of Materials

**DOI:** 10.3390/ma8085251

**Published:** 2015-08-21

**Authors:** Pascal Boulet, Damien Brissinger, Anthony Collin, Zoubir Acem, Gilles Parent

**Affiliations:** LEMTA, Université de Lorraine CNRS, 2 avenue de la Forêt de Haye - TSA 60604, Vandoeuvre les Nancy cedex 54518, France; E-Mails: damien.brissinger@univ-lorraine.fr (D.B.); anthony.collin@univ-lorraine.fr (A.C.); zoubir.acem@univ-lorraine.fr (Z.A.); gilles.parent@univ-lorraine.fr (G.P.)

**Keywords:** emissivity, absorptivity, pyrolysis, degradation, radiative transfer

## Abstract

The change in absorptivity during the degradation process of materials is discussed, and its influence as one of the involved parameters in the degradation models is studied. Three materials with very different behaviors are used for the demonstration of its role: a carbon composite material, which is opaque, almost grey, a plywood slab, which is opaque and spectral-dependent and a clear PMMA slab, which is semitransparent. Data are analyzed for virgin and degraded materials at different steps of thermal degradation. It is seen that absorptivity and emissivity often reach high values in the range of 0.90–0.95 with a near-grey behavior after significant thermal aggression, but depending on the materials of interest, some significant evolution may be first observed, especially during the early stages of the degradation. Supplementary inaccuracy can come from the heterogeneity of the incident flux on the slab. As a whole, discrepancies up to 20% can be observed on the absorbed flux depending on the degradation time, mainly because of the spectral variations of the absorption and up to 10% more, depending on the position on the slab. Simple models with a constant and unique value of absorptivity may then lead to inaccuracies in the evaluation of the radiative flux absorption, with possible consequences on the pyrolysis analysis, especially for properties related to the early step of the degradation process, like the time to ignition, for example.

## 1. Introduction

Thermal degradation involves radiative transfer as one of the key heat transfer modes, since experimental facilities generally use a strong radiative source to heat the material surface: a high temperature lamp like in the fire propagation apparatus (FPA) or a high temperature coil in a cone calorimeter. The method consists of submitting a sample to a given heat flux, calibrated before the test, which simulates the heat brought by a flame impacting on a surface in real fire conditions. Hence, the tests are performed and analyzed with reference to the value featuring the incident flux, known from a heat flux gauge measurement done at the sample center location. This raises at least two uncertainty problems related to: (i) the spectral dependency of radiation, which differs in the flame situation and in the degradation test; and (ii) to possible spatial heterogeneities in the incident flux on samples usually 100 mm × 100 mm in size. The calibration is done on the basis of a total flux, but the spectral variations of the emission are not considered. This would not be a problem for materials behaving like perfect absorbers (absorptivity equal to one) or even like grey absorbers (with constant absorptivity whatever the incident wavelength). However, real materials may absorb radiation in a selective manner, and this should be accounted for. The cone calorimeter (described in the ISO 5660 standard [1]) provides a continuous emission in the infrared range depending on the coil temperature, but with no mandatory spectral specification to our knowledge. The resulting emission is often considered close to the one of a blackbody, but deviations from a perfect emitter seem unavoidable, depending on the manufacturer or the aging of the apparatus. The case of the FPA is more complicated, since the emission is centered in the visible and the near infrared range, with sharp variations, far from the ideal emission of a blackbody [2]. If degradation occurs under flaming conditions, the radiation from the flame is also identified as a supplementary heat source, resulting in a complex and changing emission pattern as a whole. Therefore, absorptivity and emissivity become key properties for evaluating the energy absorbed by the surface or conversely the radiative heat loss by the sample. They should be known in order to evaluate the true absorbed flux and to evaluate the deviation between the degradation test under calibrated source and the material behavior under fire conditions.

This problem has to be related to the more general study of the effects due to possible errors or unavoidable uncertainties in the material properties when predicting the thermal degradation of materials. The problem of the required model complexity and the uncertainty in pyrolysis simulation was recently reviewed by Bal and Rein [3], who focused on the problem raised by a lack of knowledge of different material properties or by an over-simplification of some submodels. In particular, they concluded that a good accuracy in the degradation prediction cannot be reached without a good prediction of the energy distribution and of the heat losses. One consequence is that a good knowledge of properties is mandatory, including radiative properties, which are addressed in the present paper. Lautenberger and Fernandez-Pello [4] already presented their model Gpyro, combined with a genetic algorithm that can be used to estimate the model input parameters, including the radiative properties if not known from dedicated measurements. They acknowledged that such estimation can be obtained with significant uncertainties, and one of their conclusions was a suggestion to focus on material property estimation. Stoliarov *et al.* [5] presented various tests with the degradation model ThermaKin involving material properties, with their dependency with temperature. However, such a dependency was not applied for radiative properties due to a lack of knowledge of the absorptivity and reflectivity of the studied materials. Moreover, uncertainties up to ±50% for the absorption coefficient and ±20% for the reflectivity were reported, which shows the need for dedicated estimations with a better confidence. Finally, Linteris [6] also focused on property variations when simulating the pyrolysis of polymers with FDSand ThermaKin. The absorption of infrared radiation was included in the parameters of interest. It was observed to affect the mass loss rate and the ignition time in particular. Then, Linteris *et al.* [7] presented a paper dedicated to the role of radiative properties in the modeling of mass loss experiments, introducing a consideration for the spectral dependency of properties. Again, a potential influence was observed especially at high incident fluxes.

As numerous other parameters are also involved in the complete description of the thermal degradation process, absorptivity and emissivity can be hardly defined with their full complexity. They are generally measured or evaluated as a single parameter, defined once and for all. Such a unique value assumes that the material has a grey behavior (non spectrally-dependent) or that it can be correctly depicted by an average value (averaged in wavelength and in time despite the change in the material state). However, the material can strongly change with time during the degradation process. Forsth and Roos [8] presented an extended study on various materials, showing the complexity of the absorptivity evaluation. They concluded that its average value varies with the heat source temperature, which is a well-known consequence of a non-grey behavior. They also identified the surprising evolution of some materials with a decreasing absorptivity in the infrared range while they were `darkened’ in the visible range. This leads to the feeling that a single average value may not well represent the complex spectral nature of the sample absorptivity. Similarly Boulet *et al.* [9] showed for example that plywood, which exhibits a spectral-dependent emissivity before degradation at ambient temperature, tends toward a grey material with emissivity close to 0.90–0.95 after degradation. Moreover, the time evolution appeared to be non-monotonic, which raised the question of the possible requirement for a complex model for this property, especially if a fine description of the transient degradation is sought. The same could be said of materials like PMMA, which was extensively studied for the validation of degradation models, while its radiative behavior is of the most complex type, strongly varying with wavelength. This probably explains a part of the high dispersion of the results published on PMMA in the past, depending on the heat source used for the degradation. Recent discussions on this material can be found in [5,7,10,11,12], for example. In particular, the radiative transfer problems addressed in the present paper and a series of relevant papers on the subject were discussed in [10].

Regarding the uncertainty related to the spatial heterogeneity of the flux, the standard use of the cone ensures that the deviation in the flux does not exceed ±2% on a central square area with a 5-cm size, 2.5 cm below the cone. However, this only corresponds to 25% of the surface of a standard slab with a 10-cm size. Since the full analysis of the degradation test involves mass loss measurements, heat transfer data and smoke analysis, performed for the whole sample, the question arises whether the reference flux (measured at the sample center) is still representative of the whole test.

The present analysis was therefore conducted in order to investigate: (i) the influence of the absorptivity on the degradation of materials; and (ii) the possible error associated with the flux heterogeneity on a standard sample. The underlying idea was first to illustrate through examples how much a single value of absorptivity can result in an inaccurate evaluation of the absorbed fluxes, depending on material type, time and position. In this frame, three samples with expected different behaviors were selected: a carbon composite (near black), plywood (non-grey) and PMMA (participating medium). A comparative analysis was conducted for virgin and degraded samples to investigate how the absorptivity varies during the process and also what model complexity should be introduced for an accurate description of radiative transfer involved in the material degradation. The choice was made to consider an irradiation from a cone calorimeter in the analysis, because this is one of the most often-used apparatuses in degradation studies, but the discussion is extended to some singularities linked to the use of tungsten lamps (typical of the FPA). In the second part, simulations were conducted to evaluate the true discrepancies in the incident fluxes received near the center and at the edges of a tested sample.

## 2. Experimental Study of the Radiative Properties

### 2.1. Setup

The property $\alpha_{\nu }$ related to the spectral absorption of a material can be deduced from spectroscopic measurements. For opaque materials, the reflectivity $\rho_{\nu }$ is measured, and the property called absorptivity is its complementary part. This method was applied here on the plywood and on the carbon composite samples. For participating media, like PMMA, a transmittance measurement $\tau_{\nu }$ is also required, and $\rho_{\nu }$ is rather called the reflectance. The resulting absorption property is called absorptance, and it is deduced from the balance $\alpha_{\nu }=1-\rho_{\nu }-\tau_{\nu }$. For the present study, the required measurements were carried out using an FTIR spectrometer (Vertex 80 by Bruker) and an integrating sphere. A large number of scans were performed for the spectrum evaluations (typically up to 1000) to decrease the noise and the measurement uncertainties. Hence, the result became quite smooth, even for a fine resolution. The uncertainty itself cannot be simply evaluated, however, owing to the complexity of the measuring chain. A careful calibration with well-known samples can be done, with an apparent accuracy generally below 1%. Repeatability tests were also done for the present study, re-positioning the sample between two measurements in order to check for possible result dispersion. The repeatability was fully satisfactory (no apparent deviation between two successive measurements) for smooth samples (plywood and PMMA). For samples with heterogeneities on the surface (which is the case below for the studied carbon composite sample made with fibers and epoxy resin), average results and confidence intervals were evaluated when the deviation observed through the repeatability tests became significant.

Samples were typically studied in the range of 1000–6000 cm${}^{-1}$, with a spectral resolution of 4 cm${}^{-1}$.

The three selected samples were the following:

- A plywood sample, studied in a former study [9]. It was made of oak, its thickness was 18 mm, and its density was 580 kg/m${}^{3}$.
- A carbon composite sample, made with carbon fibers and epoxy resin, used for vessel manufacturing, with a thickness of 4.5 mm.
- A clear PMMA sample with a thickness of 15 mm.

They were chosen as representative of different typical behaviors regarding their expected interactions with radiative transfer: non-grey and opaque, or grey and opaque, or participating medium submitted to in-depth radiation.

### 2.2. Data Analysis

**Figure 1:**
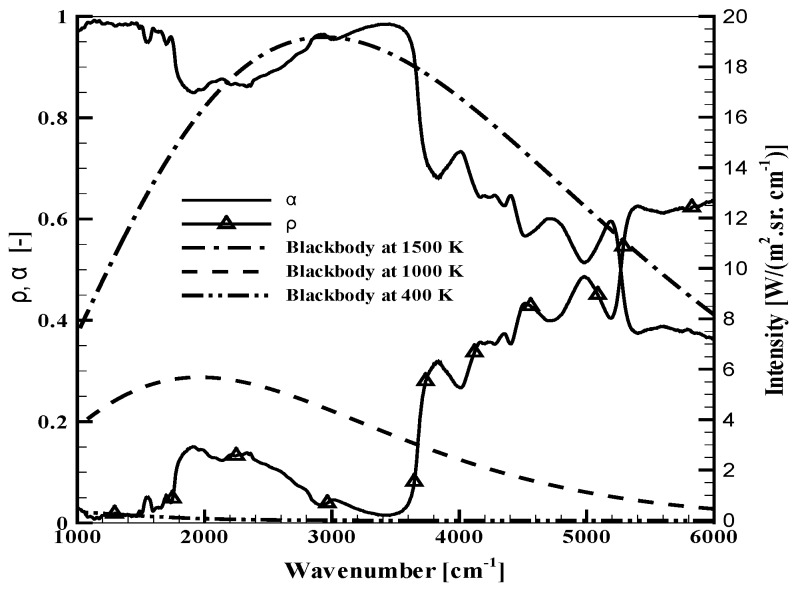
Radiative properties of the virgin plywood sample.

**Figure 2:**
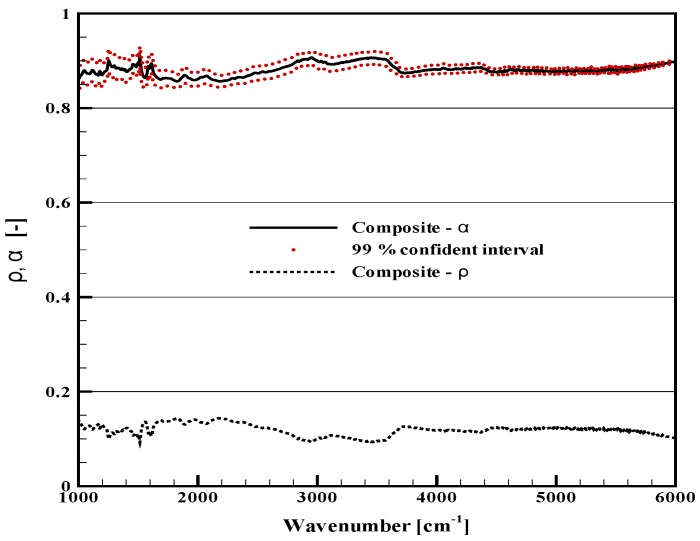
Radiative properties of the virgin carbon composite sample.

**Figure 3:**
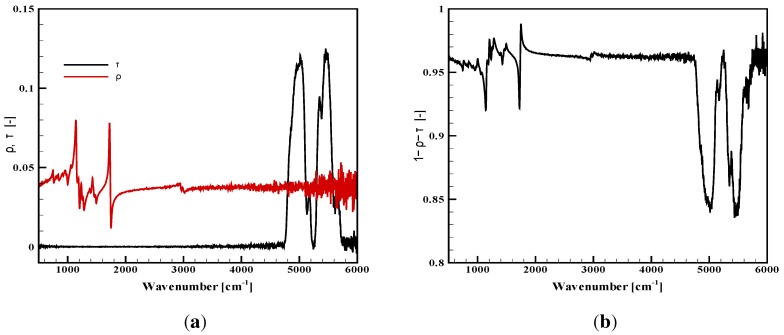
Radiative properties of the virgin PMMA slab, 15 mm thick. (**a**) Transmittance and reflectance; (**b**) absorptance.

In order to see the basic behaviors of the studied materials, results are first plotted for virgin materials in Figure 1, Figure 2 and Figure 3. They provide the properties of the samples during the very first instants of the thermal aggression, before possible alteration of the irradiated surface. For the two opaque materials (plywood and carbon composite), there is no transmission. The reflectivity and the corresponding absorptivity are simply plotted on the same graphs (Figure 1 and Figure 2). For the PMMA, data are provided for the transmittance, the reflectance and the absorptance (Figure 3a,b).

- Considering Figure 1, the plywood is obviously non-grey, as already discussed in [9]. Its absorptivity varies in a range between 0.4 and 1, being weaker in the near infrared range. It can never be considered as a black surface, and it seems that any simplification to an average absorptivity between 0.9 and 1, as it is often read in the literature, could be far from reality. Planck’s curves are also plotted in the same figure for blackbody temperatures equal to 400, 1000 and 1500 K. For a black emitter at 400 or 1000 K, the main part of the emitted radiation is below 4000 cm${}^{-1}$, in the range where the absorption variations are moderate. For a source with a 1500 K temperature, a significant emission also occurs above 4000 cm${}^{-1}$, where the non-grey behavior is obvious.
- As discussed above, the carbon composite raised some difficulties because of its heterogeneous surface, which required numerous measurements to obtain a representative average value for the reflectivity. The resulting absorptivity is presented with a confidence interval at 99% in Figure 2 (this care brought to the experimental data processing was not necessary for the two other materials with smooth and homogeneous surfaces). Considering the average properties, it can be observed that the reflectivity and the absorptivity only vary within a narrow interval. There are some spectral variations, but these are obviously limited, and in this case, the grey assumption could be considered with less inaccuracy than for the plywood. The carbon composite sample cannot be simplified as a black surface, but a representative value can be given for an average absorptivity, as will be discussed below.
- For the virgin PMMA, in-depth radiation is observed, and the analysis rather leads to the identification of an absorptance or an absorption coefficient, which represents the volumetric absorption of radiation inside the sample, rather than an absorptivity, which would correspond to a surface property. A presentation of the absorptance was chosen here, but details on the derivation of an absorption coefficient can be found in [11]. Figure 3a presents the sample transmittance and reflectance, while Figure 3b shows the corresponding absorptance. These properties were compared to a similar work carried out on other samples of PMMA [11] (not shown here), showing the perfect agreement with the properties obtained during the present study. As for the plywood, the sample exhibits a non-grey behavior. In the case of the PMMA, it is mainly a consequence of some variations in the transmittance for high wavenumbers. The two sharp transmission peaks close to 5000 cm${}^{-1}$ would be even higher for thinner samples. The absorptance of the present 15 mm-thick sample ranges between 0.84 and 0.99. The influence of this non-grey behavior will depend on the spectral distribution of the radiative source used for the sample heating. As a cone calorimeter is considered here, it will mainly depend on the coil temperature, and the same comments related to the Planck’s curves could be done as for Figure 1. PMMA would appear especially non-grey for sources with high temperatures. Note that the absorptance is the result of the global sample response to the radiation, but the in-depth radiation also changes with the wavenumber, meaning that radiation would reach different depths inside the sample depending on the wavenumbers. Hence, the sample heating occurs in the material volume at various positions.

**Figure 4:**
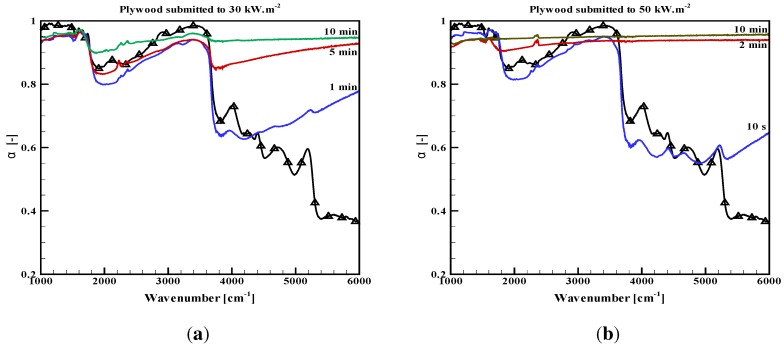
Absorptivity of a plywood sample submitted to thermal degradation during prescribed times. (**a**) Incident flux 30 kW/m${}^{2}$; (**b**) incident flux 50 kW/m${}^{2}$.

**Figure 5:**
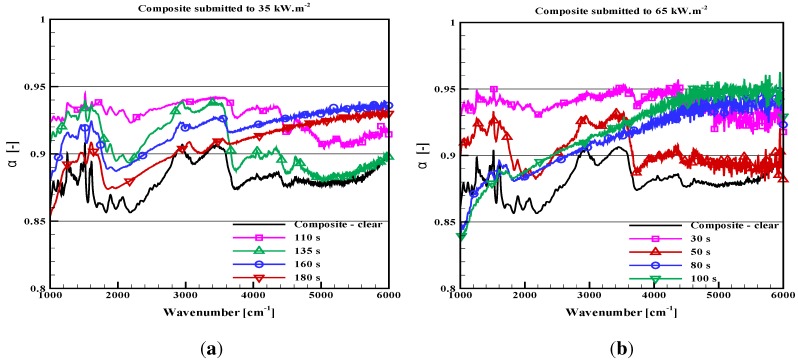
Absorptivity of a carbon composite submitted to thermal degradation during prescribed times. (**a**) Incident flux 35 kW/m${}^{2}$; (**b**) incident flux 65 kW/m${}^{2}$.

**Figure 6:**
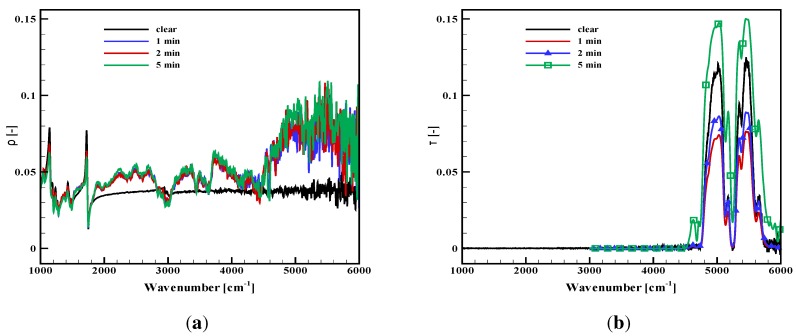
Reflectance, transmittance and absorptance evolution of a 15 mm-thick PMMA sample submitted to thermal degradation with an incident flux of 50 kW/m${}^{2}$ during prescribed times. (**a**) Reflectance; (**b**) transmittance.

As the degradation occurs, the absorptivity of the present opaque samples and the in-depth absorption of the PMMA change with time. This is illustrated through Figure 4 and Figure 5 for the plywood and the carbon composite sample. The absorption properties are plotted after a thermal aggression with given radiative fluxes (typically 30, 50 or 65 kW/m${}^{2}$) during different definite exposition durations (up to 10 min). For the PMMA, the reflectance and the transmittance are provided for an incident flux of 50 kW/m${}^{2}$ (Figure 6a,b). Measurements for PMMA were also carried out for a 30-kW/m${}^{2}$ incidence, but they are not plotted here, since the observed tendencies were the same, only with a slightly slower evolution. It must be noted that all of these results were not obtained *in situ*, at high temperature, but on cold samples withdrawn from the cone calorimeter after a prescribed duration and cooled down to ambient temperature. Consequently, the absorption properties may be slightly different from the true sample properties at high temperature during a thermal degradation test (with no possibility to evaluate this deviation). However, the observed tendencies of the spectral evolution or the global variation are expected to be representative of the materials. The objective of our analysis is focused on these tendencies rather on absolute values.

- The plywood absorptivity globally increases in the near-infrared range (Figure 4). It tends to a grey behavior with an absorptivity above 0.90, after an exposure duration, which of course depends on the incident flux, as already commented on in [9]. The higher the incident flux is, the faster the transition to grey behavior is, but it always lasted a few minutes for the present studied fluxes. There are some strange local decreases in some wavenumbers, which were explained through complex internal multiple reflections within the carbon layer developing at the sample surface in [9]. However, as a whole, the grey assumption becomes more and more valid as the degradation process occurs.
- The carbon composite absorptivity also slightly varies, but the scale in Figure 5 is modified, such that the variations are actually not so important. Recalling that the repeatability for this sample is not as good as for the other samples, the variations are in fact within a narrow range between 0.85 and 0.95. The first increase is observed up to 0.95, followed by a slight decrease, but still at high values close to 0.90. The complex degradation process probably involving the resin decomposition first and the carbon fibers aggression in the second step, could explain these variations. In any case, the grey assumption seems to be acceptable during the whole degradation test.
- The measurements for PMMA are still presented through the slab reflectance and transmittance (Figure 6). The reflectance seems to quickly evolve to a slightly higher value in the near-infrared range, especially around 5000 cm${}^{-1}$ and with no more change below 1800 cm${}^{-1}$. The changes are weak, since the spectral variations are limited between 0.03 and 0.10, but at the same time, the sample aspect is clearly affected and the sample less translucent. Similarly, the transmittance is varying in the near-infrared, in the range corresponding to the peaks observed on the virgin material close to 5000 cm${}^{-1}$. The first decrease in the transmittance is observed for an irradiation during one minute as a consequence of an opacification, which is obvious through visible observation. Then, the transmittance strangely re-increases, but this can be explained by the sample thickness regression, since in-depth absorption occurs in a slab with decreasing thickness. The transmittance is still lower than the one measured on virgin samples with similar thicknesses [11]. This is due to an opacification of the degraded sample as compared to a virgin sample, which is obvious in the visible range and also appears here in the infrared. For a longer time exposure, the sample alteration is so important that the sample shape itself is altered and the measurements cannot be done anymore. As a whole, increases in the reflectance and transmittance values modify the absorptance toward stronger spectral variations (Figure 6c). For a short time exposure, the decrease in transmittance first compensates the increase in reflectance. However, for a longer time exposure, the absorption decreases, in particular in the high wavenumbers, down to 0.75. Note that the modification is moderate below 4000 cm${}^{-1}$. This leads us to the conclusion that the consequences on the absorbed flux will not be important when the sample will be submitted to a cone calorimeter or any source with a temperature below 1000 K (the main part of the emission below this 4000 cm${}^{-1}$ threshold), but it could be dramatic for high wavenumber sources, like tungsten lamps used in an FPA.

In order to evaluate the changes in absorption quantitatively, average properties can be computed applying Planck’s averaging as follows:

(1) $$\alpha =\frac{\int \alpha_{\nu }I_{b\nu }\left(T\right)d\nu }{\int I_{b\nu }\left(T\right)d\nu }$$

where $I_{b\nu }\left(T\right)$ stands for the intensity of a blackbody at temperature *T* and should be replaced by the intensity of any irradiation source used for the thermal aggression. Note that this formulation also shows that the average surface emissivity should not be taken as equal to the average surface absorptivity, as is usually done in the literature, unless if the reference temperatures used for the average computations (*i.e.*, the temperature of the irradiation source for the computation of the average absorptivity and the sample surface temperature for the computation of the average emissivity using the same relationship only replacing $\alpha_{\nu }$ with $\epsilon_{\nu }$) are the same, or if the sample is grey (constant property with wavenumber, meaning that it can be put outside the integrals).

Of course, such averages are only fully representative of the material properties for samples with near grey behaviors, while hiding the problem of the spectral variations for non-grey materials. However, it will help to evaluate the requirement for an update of the radiative properties as the degradation occurs or conversely it will show if constant properties can be simply used.

Average absorption properties are given in the two first columns of Table 1. They were computed for sources assumed to be black at temperatures of 1000 and 1500 K. All values are in the range between 0.78 and 0.96, which indicates that the present samples are all good absorbers of the incident radiative flux. When the samples are submitted to thermal degradation, their absorptivity systematically increases above 0.90 after 2 min. For the samples with the highest absorption, some slight decreases may occur for a long time exposure, but always with the absorptivity remaining above or close to 0.90. Hence, this table shows that common practices, which lead researchers to guess that the absorptivity of degraded materials is between 0.9 and 1, are not so bad. The only exception is for the PMMA after significant degradation, since a value down to 0.87 is found after five minutes under 50 kW/m${}^{2}$. In this case, the thickness decrease explains the phenomenon as the in-depth absorption occurs in a thinner and thinner sample. Of course, these average tendencies should not hide spectral discrepancies, especially occurring for high wavenumbers. The influence of the source temperature is weak. The virgin plywood is the only sample for which a difference in the absorptivity is observed depending on the source temperature. Actually, this is the sample with the most significant non-grey behavior in the present study. As the degradation leads the samples toward grey behaviors, average absorptivities are no longer varying with the source temperature after significant degradation. Remember that most of the emission considered here is in the infrared range below 5000 or 6000 cm${}^{-1}$. An effect could still appear for significantly higher source temperatures or considering any source with significant emission in the near-infrared range and the visible (like the tungsten lamps of an FPA). The question also arises of the influence of the flame developing along the sample after ignition. Such a flame is often optically thin, with sharp peaks in ranges due to CO${}_{2}$ and H${}_{2}$O absorption, which are mainly above 4000 cm${}^{-1}$. This would require a dedicated analysis, but the properties weighted with the corresponding spectral intensity would probably be close to those listed in Table 1 and commented on above.

**Table 1:** Average absorptivity or emissivity computed between 1000 and 6000 cm${}^{-1}$, α for two high source temperatures of 1000 K, 1500 K and ϵ for a moderate temperature of 400 K.

Material	α at *T* = 1000 K	α at *T* = 1500 K	ϵ at *T* = 400 K
Plywood - virgin	0.86	0.78	0.95
Plywood - 30 kW/m${}^{2}$, 1 min	0.85	0.80	0.92
Plywood - 30 kW/m${}^{2}$, 5 min	0.90	0.90	0.93
Plywood - 30 kW/m${}^{2}$, 10 min	0.94	0.94	0.95
Plywood - 50 kW/m${}^{2}$, 10 s	0.84	0.77	0.93
Plywood - 50 kW/m${}^{2}$, 2 min	0.93	0.93	0.93
Plywood - 50 kW/m${}^{2}$, 10 min	0.95	0.95	0.94
Carbon composite - virgin	0.88	0.88	0.86
Carbon composite - 35 kW/m${}^{2}$, 110 s	0.93	0.93	0.93
Carbon composite - 35 kW/m${}^{2}$, 135 s	0.91	0.91	0.91
Carbon composite - 35 kW/m${}^{2}$, 160 s	0.91	0.91	0.89
Carbon composite - 35 kW/m${}^{2}$, 180 s	0.89	0.89	0.86
Carbon composite - 65 kW/m${}^{2}$, 30 s	0.94	0.94	0.94
Carbon composite - 65 kW/m${}^{2}$, 50 s	0.91	0.91	0.91
Carbon composite - 65 kW/m${}^{2}$, 80 s	0.89	0.91	0.85
Carbon composite - 65 kW/m${}^{2}$, 100 s	0.90	091	0.84
PMMA - virgin	0.96	0.95	0.96
PMMA - 30 kW/m${}^{2}$, 1 min	0.92	0.95	0.96
PMMA - 30 kW/m${}^{2}$, 2 min	0.93	0.95	0.96
PMMA - 30 kW/m${}^{2}$, 5 min	0.92	0.95	0.96
PMMA - 50 kW/m${}^{2}$, 1 min	0.92	0.94	0.96
PMMA - 50 kW/m${}^{2}$, 2 min	0.92	0.94	0.96
PMMA - 50 kW/m${}^{2}$, 5 min	0.87	0.93	0.96

The fourth column in Table 1 was obtained considering a Planck’s average for a lower reference temperature equal to 400 K, which is more representative of an emissivity value for the surface after moderate heating. Therefore, the cases with short degradation times are more representative of realistic values, since we can expect that the temperature level would be higher than 400 K for longer time exposures. It can be seen that the emission and the absorption properties can differ significantly, especially for the non-grey materials like plywood. PMMA is also non-grey, but not in the weak wavenumber range, which explains why absorptivity and emissivity are not affected by the present averages, except when the sample thickness changes, after significant degradation.

As a summary, when introducing a constant absorptivity in a degradation model, two error sources should be kept in mind: (i) for non-grey samples (sometimes with more pronounced spectral variations than the PMMA and the plywood taken as illustrations here), an average value may penalize the accuracy of the real absorbed flux, depending on the used radiative source; and (ii) the absorptivity currently reaches values as high as 0.90 to 0.95, but its value may be significantly smaller at the early stage of the degradation process because of the spectral dependence or after a significant degradation duration because of sample regression for materials with in-depth absorption, such that a unique value does not provide the true absorption history of the material. In particular, a property like the time to ignition, which is representative of the early step of degradation, is probably sensitive to the inaccuracies in the absorbed flux.

## 3. Radiative Flux Simulation

**Figure 7:**
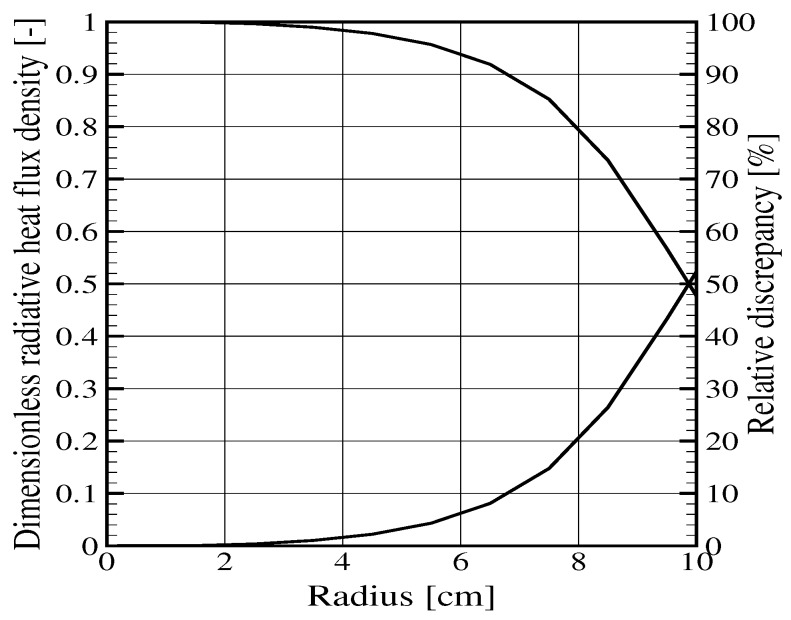
Numerical evaluation of an incident flux received under a cone, at a distance of 2.5 cm below the cone. Normalized flux density (left-hand scale) and relative discrepancy with the maximum value (right-hand scale) as a function of the radial distance from the cone axis.

The previous section showed that the knowledge of the absorptivity values and their evolutions as the degradation process goes on directly affect the prediction of the radiative flux absorption. However, a supplementary inaccuracy can come from the spatial distribution of the incident flux. Still focusing on the cone calorimeter, despite the care brought to these standard tests, some heterogeneities in the incident flux occur on the sample. In order to evaluate the discrepancies, one can compute the flux received on every point of a sample. For the present investigation, a ray tracing approach was conducted as presented in [2], introducing a realistic geometry for the cone and considering an emission from a blackbody at a given constant temperature corresponding to a given set point of a cone. Details of the geometry are taken from a real cone, as described in [2]. The main dimensions are recalled here: the exact helicoidal coil is represented by 10 tores with a progressive increase in the diameter. The largest tore has a mean diameter of 19 cm, the smallest one 5.5 cm, and the height of the device is 8 cm. All tores are supposed to have the same temperature, which is an idealization of the true situation, because previous measurements have shown that a real cone may present some heterogeneities on its emitting surface (see [9] for example). All of the results were obtained based on the tracking of 10${}^{10}$ rays/m${}^{2}$ of the emitting surface. In order to ensure that the convergence was reached, a specific test with 10${}^{11}$ rays/m${}^{2}$ for one element of the geometry showed a discrepancy on the predicted fluxes below 0.1% as compared to the simulation with 10${}^{10}$ rays/m${}^{2}$. Figure 7 presents the flux received on a central line located on a horizontal surface 2.5 cm below the cone (the usual position of the sample in standard conditions), as a function of the radial position with the origin on the cone axis. In order to present results independent from the choice of the cone temperature, the radiative flux density is normalized dividing the local flux by the maximum flux value, computed at the sample center along the cone axis. The flux has a maximum value at the center and decreases progressively toward the edge of the cone. A second curve provides the discrepancy in % as compared to the maximum value (right-hand scale). It can be seen that the deviation from a constant flux is very low below the radial value of 2.5 cm (the deviation is below the ± 2%, which is the requirement stated in the ISO 5660-1 standard). Of course, the discrepancy quickly increases with the radial position, but standard tests usually concern samples with a size of 10 cm, centered along the cone axis, such that the maximum discrepancy never reaches the maximum value of 50% seen in Figure 7. With a square sample 10 cm × 10 cm, the relative discrepancy on the edge would be between 4% (at the middle of the edge, corresponding to a radius of 5 cm) and around 10% on the corners (radius above 7 cm).

**Figure 8:**
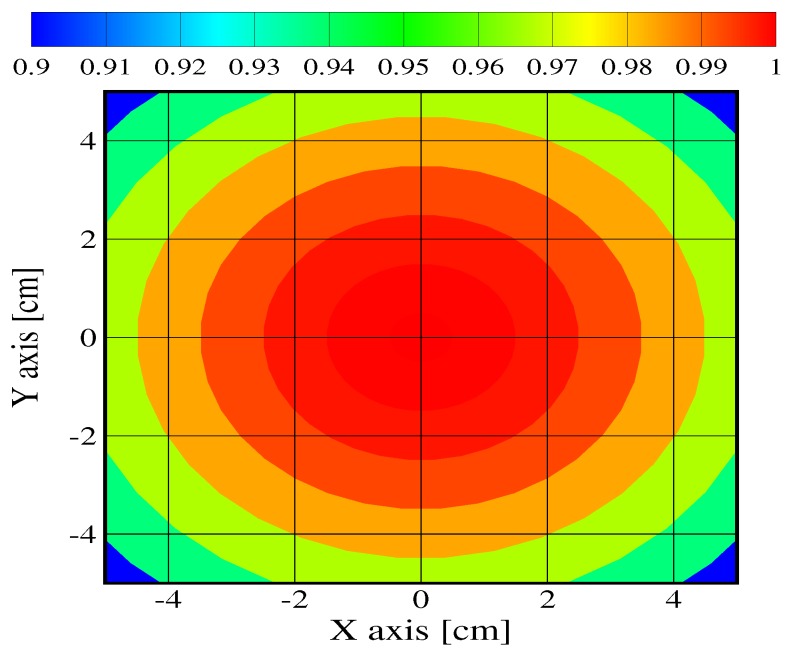
Contours of normalized radiative flux density on a 10 cm×10 cm sample located at 2.5 cm below the cone.

This is confirmed in Figure 8, which presents the contours of incident flux for a sample with surface 10 cm × 10 cm. Data are still normalized, such that results would hold for any set point in terms of incident flux, provided that the cone can be assumed isothermal. Any deviation from constant temperature due to cone aging or design could increase the discrepancies in the flux contours. In the present ideal case, a wide central area obviously ensures a constant flux, but discrepancies up to 10% are obtained on the corners. To our knowledge, such a problem is not usually accounted for in the models. Data are provided for the degradation of the materials assuming that a constant prescribed flux is received from the cone, whatever the degradation duration and the location on the sample.

It must be understood from the previous data analysis that for the plywood (the sample with the most spectacular absorption variations), a heat flux set point at 50 kW/m${}^{2}$ would result in an absorbed flux around 40 kW/m${}^{2}$ at the sample center when the test begins (assuming an absorptivity close to 0.8 for a cone temperature in the range between 1000 and 1500 K) decreasing down to 36 kW/m${}^{2}$ near the corners. During the degradation, these values would increase up to 47.5 kW/m${}^{2}$ and 42.7 kW/m${}^{2}$, respectively (assuming an absorptivity close to 0.95). The maximum deviations in the absorbed flux are around 19% in time and 10% in space. For the other samples, the variations would be less spectacular (around 10% as a whole).

Note that this comment does not consider the incident flux modification due to the flame generated by the pyrolysis products. Deviations from the set flux have been already attributed to the flame developing along the sample, depending on the materials in particular, but what is seen here is that the contribution of the heater to the absorbed flux itself may also vary significantly with time, position and wavelength.

## 4. Conclusions

A study was carried out on the deviations of the absorbed flux as compared to the unique set point often used as a reference during degradation tests.

A first important cause of error is the spectral response of the material to the incident flux. Materials may have different behaviors, but non-grey samples in particular can absorb fluxes with significant discrepancies depending on the source. An average absorption may hide this difficulty, with two consequences rarely accounted for: (i) a time-varying absorption because most samples tend to a grey behavior during degradation tests; and (ii) an emissivity different from the absorptivity as the reference temperatures used for averaging the absorptivity/emissivity are not the same. In the present tests, variations up to 20% were found, for example, between the properties of a virgin plywood and a degraded plywood (the sample with the most variable spectral behavior). Most of the tested samples showed an absorptivity or an absorptance in the range between 0.90 and 0.95 after significant degradation duration, but this hides an early step of degradation possibly with different values, which may affect the prediction of the time to ignition among others.

A second error cause is the heterogeneous incident flux on the sample, demonstrated in the present work for a degradation test under a cone calorimeter. Deviations up to 10% were found between the center and the edges of a standard sample with dimensions 10 cm × 10 cm.

Finally, another significant problem, possibly overwhelming the two previous on some materials, is the supplementary flux caused by the flame after ignition of the sample. This point could receive more attention in an extension of the present work.

All of these uncertainties should be kept in mind when modeling the degradation and when investigating the sensitivity of the degradation results to the incident radiative flux.

One series of suggestions to modelers studying thermal degradation could be to ask for a systematic measurement of the sample reflectivity in a range between 1000 and 6000 cm${}^{-1}$ before any simulation. In case of a semi-transparent medium, transmissivity measurement could be also done for an estimation of the absorption coefficient involved in a model for in-depth radiation. If significant spectral variations are observed, Planck’s averages should be computed at different reference temperatures in order to bracket the variations of the absorptivity and the emissivity. One should keep in mind that the absorptivity often tends toward the above-mentioned [0.90–0.95] range after significant degradation and that this value should be updated with time if the starting value is significantly smaller (the case of weakly absorbing materials). Then, a sensitivity study of the radiative properties should be conducted, varying the values in a range of ±20%, owing to the present observations. Finally, a reduction of the sample size to 8 cm × 8 cm could be a simple way to reduce significantly the spatial inhomogeneities in the incident flux (the maximum deviation would fall down to 4% instead of 10%).
